# Striking diflubenzuron resistance in *Culex pipiens*, the prime vector of West Nile Virus

**DOI:** 10.1038/s41598-017-12103-1

**Published:** 2017-09-15

**Authors:** Linda Grigoraki, Arianna Puggioli, Konstantinos Mavridis, Vassilis Douris, Mario Montanari, Romeo Bellini, John Vontas

**Affiliations:** 1 Institute of Molecular Biology and Biotechnology, Foundation for Research and Technology-Hellas, 73100 Heraklion, Greece; 2 Department of Biology, University of Crete, Heraklion, 70013 Greece; 3 Medical and Veterinary Entomology, Centro Agricoltura Ambiente “G. Nicoli”, Bologna, Italy; 4 Azimut, Ravenna, Italy; 5 Department of Crop Science, Pesticide Science Lab, Agricultural University of Athens, 11855 Athens, Greece

## Abstract

*Culex pipiens* mosquitoes cause severe nuisance and transmit human diseases including West Nile. Vector control by insecticides is the main tool to prevent these diseases and diflubenzuron is one of the most effective mosquito larvicides used in many places. Here, high levels of resistance were identified in *Cx*. *pipiens* from Italy, with a Resistance Ratio of 128 fold. The phenotype was associated with mutations at amino acid I1043 (I1043M and I1043L) of the Chitin synthase gene, which showed significantly higher frequency in bioassay survivors. Both mutations have been introduced in the *Drosophila melanogaster* chitin synthase gene using the genome editing method CRISPR/Cas9 and validated to confer significant levels of resistance, although at different levels. The I→M mutation results in a Resistance Ratio >2,900 fold and the I→L mutation >20 fold. Two PCR based diagnostics were developed for monitoring of the resistant mutations in field populations. The findings are of major concern for public health given the importance of diflubenzuron in mosquito control in many places, the intensity of the resistance phenotype and the limited availability of alternative larvicides.

## Introduction

Members of the *Culex pipiens* complex are among the most common mosquitoes in urban areas. In addition to biting nuisance, they are also the main vectors of several arboviruses, such as West Nile Virus (WNV), St. Louis encephalitis (SLE), Sindbis (SINV), Rift Valley fever (RVFV) and Japanese encephalitis (JEV)^1^, as well as primary vectors of lymphatic filariasis^2^.

Vector control is the main tool to prevent diseases transmitted by mosquitoes, particularly in the absence of effective vaccines, as is the case for all of the above pathogens except of JEV. A key part of these programs is the reduction of the vector’s population size by chemical control. This includes the use of mosquito adulticides either indoors or outdoors and application of larvicides to breeding sites. The latter are used extensively for the control of *Cx*. *pipiens* mosquitoes in urban and semi-urban environments, where the larval habitats are largely present. However, a limited number of mosquito larvicides are available,: (i) temephos, an organophosphate which has been used worldwide for several decades, is currently no longer available in Europe; (ii) microbial bacterial toxins *Bacilus thuringiensis israelensis* (Bti) and *Bacillus (Lysinibacillus) sphaericus* (Bs), currently used extensively against mosquito larvae in many regions, although their efficiency may be sometimes affected by certain ecological conditions^3,4^; (iii) hormonal insect growth regulators (methroprene and pyriproxyfen), which have been used to a lesser extent and (iv) the chitin biosynthesis inhibitor diflubenzuron, currently one of the most effective mosquito larvicides in Europe and other regions.

Diflubenzuron is a member of the Benzoyl(phenyl)urea family (BPUs-Group 15 based on the IRAC grouping system)^5^. It is an environmentally friendly insecticide that inhibits the chitin biosynthesis process affecting the insect’s growth. A recent study revealed the precise mode of action of BPUs including diflubenzuron, which is the direct interaction with chitin synthase 1^6^.

The progressive reduction in the number of insecticides available for use in Public Health and their intense use can result in the rapid selection of insecticide resistance. Resistance development in *Cx*. *pipiens* mosquitoes has not proceeded at the pace observed in other vectors, such as *Anopheles* and *Aedes* species^7,8^. However, a number of low-moderate resistance cases have been reported, against pyrethroids^9^, temephos^10^, as well as Bacillus toxins^11–13^.

Insecticide resistance mechanisms involve mutations at the target site of insecticides, which render them less sensitive to inhibition by decreasing their affinity for the insecticide molecules, as well as detoxification enzymes, which metabolize or sequester the insecticide molecules keeping them away from their target^14^. Pyrethroid and organophosphate resistance have been associated with both target site mutations and detoxification enzymes in *Cx*. *pipiens*^9,15^, while pyriproxyfen resistance has been associated with the upregulation of P450 oxidases in other mosquito species^16^.

Diflubenzuron resistance has not been found yet in mosquitoes. However, diflubenzuron resistance was recently reported and characterized in the major agricultural pest *Plutella xylostella* (Lepidoptera), where a mutation on the C-terminal region of chitin synthase, namely I1042M (*P*. *xylostella* numbering), was associated with striking levels of resistance^6^.

Here, we report the identification of field caught *Cx*. *pipiens* populations with high levels of resistance against diflubenzuron and the presence of mutations strongly associated with the resistance phenotype. Given the limited availability of alternative larvicides, the findings are of major concern for public health.

## Results

### Diflubenzuron bioassays

Two field populations were collected in 2015 and 2016 from Ravenna, Italy, an urban area where diflubenzuron had been used exclusively for mosquito control for several years with approximately 40-60 diflubenzuron based treatments, and tested for their susceptibility to diflubenzuron (Table 1). The “Ravenna 2015” population showed considerable resistance to diflubenzuron (RR_LC50_ = 32 fold), which increased after one year (“Ravenna 2016”) and approximately 5 diflubenzuron field applications, to RR_LC50_ = 128 fold, in 2016, exceeding the recommended WHO dosage of diflubenzuron in potable water containers (0.25 ppm)^17^. The estimated LC_90_ of the Ravenna population in 2016 is 8.51ppm, dramatically higher than the recommended WHO dosage, indicating a likely significant operational impact of resistance.

**Table 1:** Log-dose probit-mortality data for diflubenzuron tested against third-fourth instar larvae of *Culex pipiens*. *Number of larvae tested.

Mosquito population	N*	LC_50_ (95%CL)^†^	RR_LC50_^‡^	Χ^2^ (df)^§^
Benaki 2013 (lab strain)	120	0.002 (0.001–0.003)	1	8.52 (10)
Ravenna 2015	600	0.065 (0.026–0.396)	32.5	16.838 (22)
Ravenna 2016	600	0.257 (0.146–0.619)	128.5	11.860 (10)

^†^Lethal concentration 50 in mg L^−1^ s± 95% Confidence Limits. ^‡^Resistance Ratio (calculated over the Benaki strain). ^§^Chi-square testing linearity of dose-mortality response with degrees of freedom.

### Identification of diflubenzuron resistance mutations

**Figure 1:**
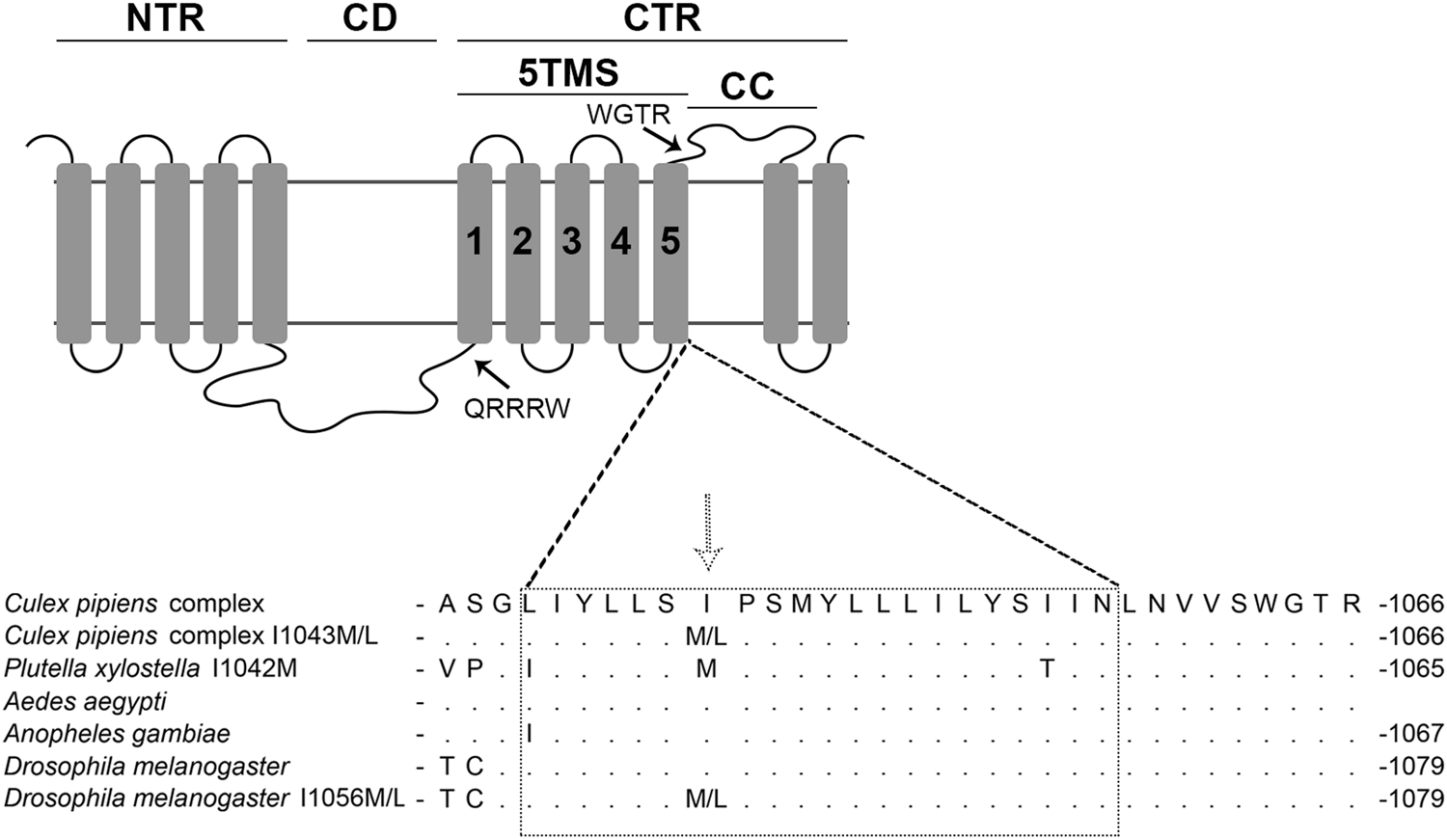
Schematic representation of the Chitin Synthase and the position of the diflubenzuron resistance mutations. NTR, N-terminal region; CD, catalytic domain; CTR, C-terminal region; 5TMS, cluster of five transmembrane segments; and CC, coiled-coil motif. Rectangular boxes represent transmembrane domains. Arrows point to signature sequences QRRRW (catalytic domain) and WGTR (N-terminal region). Lower part: Aligned amino acid sequences of the fifth helix of the 5TMS (gray rectangular) from different insects. An arrow points to the position I1043 (*Cx*. *pipiens* numbering), where the diflubenzuron resistance mutations I1043M and I1043L are found (adapted from ref.^25^).

A sequence of 825 bp, part of the chitin synthase C-terminus, the putative binding site of diflubenzuron, spanning the 1043 position (*Cx*. *pipiens* numbering) was amplified from *Cx*. *pipiens* gDNA (Fig. 1) and examined for the presence of mutations. All individuals from the reference laboratory susceptible strain (Benaki) were homozygous for the wild type IIe (I) amino acid, i.e. the presumed susceptible allelic form^6^. However, 9 out of 34 individuals tested (26.4%) from the most resistant Ravenna 2016 population (RR_LC50_ 128 fold), carried alleles with mutations at the 1043 site, either the previously characterized I1043M (I1042M based on *P*. *xylostella* numbering)^6^ or the novel I1043L. More specifically, 4 individuals were homozygotes for the mutated alleles (I1043M/I1043M or the combination I1043M/I1043L) and 5 were heterozygotes (I1043/I1043M or I1043/I1043L) (Table 2). The frequency of mutated alleles increased significantly (P value <0.0001, Fisher's test) in the bioassay survivors of the Ravenna population (bioassay doses 0.0187–0.468 ppm): 17 individuals out of 30 tested were homozygous for the mutation I1043M, 13 individuals were homozygous for the I1043L mutation and none had the susceptible allelic form, not even in heterozygote state.

**Table 2:** Screening of *Culex pipiens* individuals for the presence of mutations at the I1043 site of the chitin synthase.

Population	Sample size* (N)	Genotype at the chitin synthase position 1043					
		Homo^†^ (II)	Homo ^‡^(MM)	Homo^§^ (LL)	Hetero(IM)	Hetero(IL)	Hetero(ML)
Benaki 2013	14	14	—	—	—	—	—
Ravenna 2016	34	25	3	—	2	3	1
Ravenna 2016 (Survivors)	30	—	17	13	—	—	—

*Number of individuals tested. ^†^I:Isoleucine amino acid (wild type). ^‡^M: Methionine amino acid. ^§^L:Leucine amino acid.

### Functional characterization of the I1043L mutation

Embryos of the nos.Cas9 *Drosophila* strain were injected with a gRNA/donor plasmid mix and their progeny was screened in pools for the presence of genome-modified alleles. Out of the 29 flies (G_0_) that gave progeny, 12 were positive for the presence of genome modified alleles in G_1_ pools. G_1_ individuals from the 12 different original G_0_ lines were outcrossed to the yw strain and then screened to test whether they carried the genome modified allele. G_2_ individuals from positive G_1_ parents derived from five original (G_0_) lines were selected for generating homozygous lines by crossing to balancer flies and screening to identify positive heterozygotes. Several independent homozygous lines were established after balancing and verified by sequencing to be homozygous for the I1056L (equivalent to the I1043L in *Cx. pipiens*) allele. One of these, the Dif4 line was used to perform toxicity assays with diflubenzuron.

Toxicity assays with diflubenzuron were conducted using the Dif4 line and the parental nos.Cas9 strain as a susceptible control. The previously established diflubenzuron resistant Px39 line harboring mutation I1056M^6^ was also used, as a positive control. The LC_50_ value obtained for the nos.Cas9 strain possessing the wild type allele was 0.34 ppm, while the LC_50_ for the Dif4 line was 7.52 ppm resulting in a resistance ratio of approximately 22-fold (Table 3). The positive (resistant) control strain Px39 did not show any mortality even at the 1,000 ppm dose, which was the highest dose tested in this study.

**Table 3:** Bioassay results with diflubenzuron in genome-modified *Drosophila*.

Strain	LC_50_ in ppm (95% CL)	Resistance Ratio (95% CL)
nos.Cas9	0.34 (0.31–0.37)	1
Dif4 (I1056L)	7.52 (6.73–8.19)	22.1 (19.7–24.5)
Px39 (I1056M)	>1,000	>2,941

LC_50_ values (in ppm, mg/L) for diflubenzuron are shown for the control flies (nos.Cas9) and the genome modified flies Dif4 (I1056L) and Px39 (I1056M) with the respective 95% confidence limits. The resistance ratio is calculated over the control flies.

### Diagnostic assays

**Figure 2:**
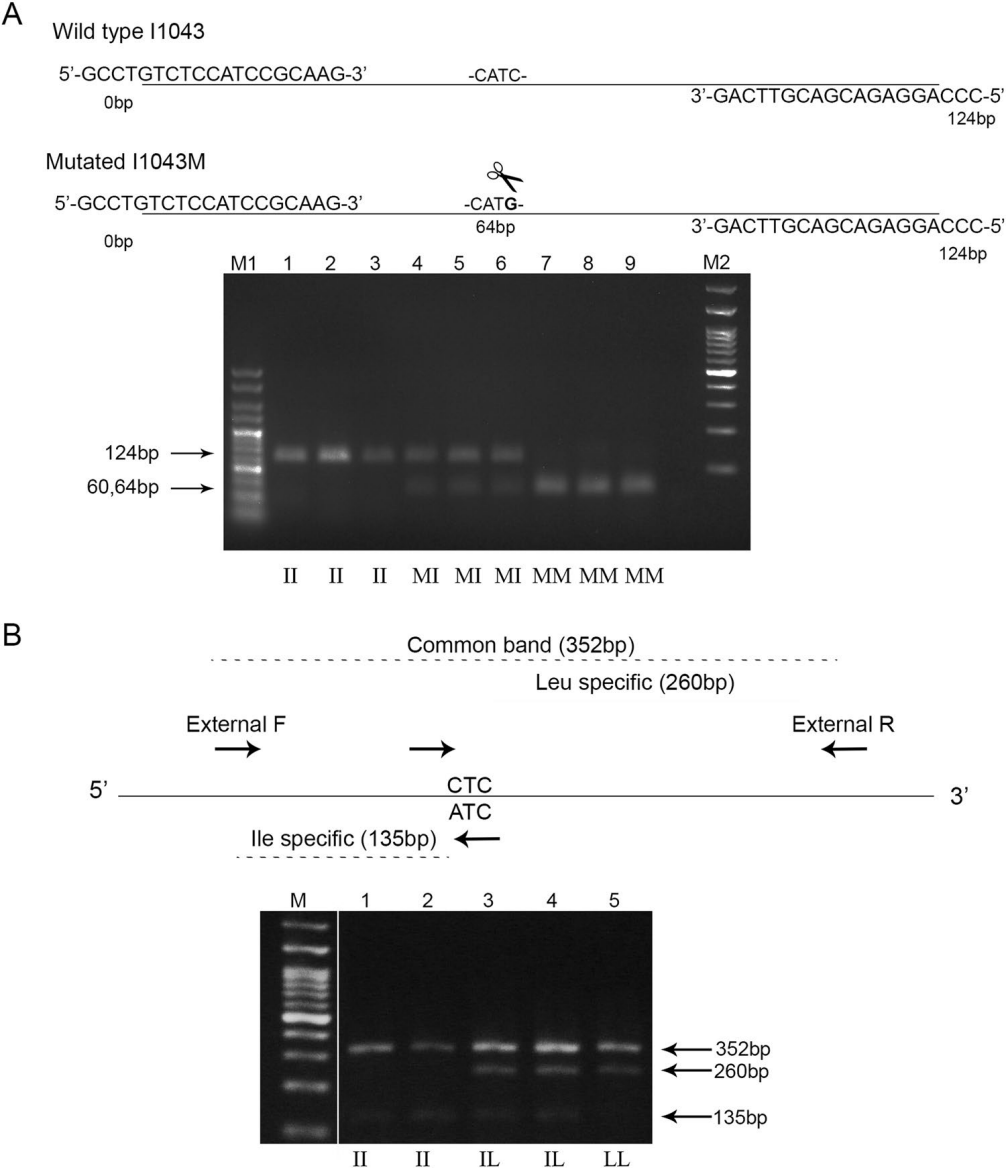
Molecular diagnostic assays for the detection of diflubenzuron resistance mutations in *Culex pipiens*. (**A**) Diagnostic PCR-RFLP for the mutation I1043M. Upper part: Schematic representation of the assay. The position and sequence of primers used to amplify the 124 bp fragment are presented. The position of the NlaIII restriction site created by the mutation (CATC → CAT**G**) is depicted as a pair of scissors at the middle (64 bp) of the PCR product. Below is shown a representative agarose gel (3%) were the NlaIII digested PCR products are shown. M: HyperLadder V 25pb (Bioline,UK), Lanes 1, 2, 3 individuals homozygous for the wild type allele (II), Lanes 4, 5, 6 individuals heterozygous for the mutated allele (IM), Lanes 7, 8, 9 individuals homozygous for the mutated allele (MM), M2 100 bp DNA ladder. (**B**) Allele specific PCR for the mutation I1043L.Upper part: Schematic representation of the assay. The position of the four primers used is shown. External_F and External_R primers pair and produce a common 352 bp band. External_F and ATC_ R pair and produce a susceptible (Ile) specific 135 bp band and CTC_F and External_R pair and produce a resistant (Leu) specific 260 bp band. Lower part: Representative agarose gel (2%) with obtained PCR products. M 100 bp ladder, Lane 1, 2 products from homozygous susceptible individuals (II), Lanes 3, 4 products from heterozygous individuals (IL) and Lane 5 product from a homozygous mutated individual (LL). White line denotes the part of the gel that has been cropped and re-grouped. Full-length gels are presented in Supplementary Fig. S1.

A PCR-RFLP diagnostic assay using the NlaIII endonuclease (5′-CATG-3′) was developed to identify the I1043M diflubenzuron resistance mutation at the C-terminus of the *Cx pipiens* chitin synthase. The PCR product (124 bp) from the susceptible allele or any other allele without the I1043M mutation is not cleaved by the enzyme. In contrast the I1043M mutation (ATC → ATG) creates a restriction site at the middle (64 bp) of the amplified product producing two fragments of 60 and 64 bp respectively. Thus, homozygous susceptible individuals show a single intact band of 124 bp, homozygous for the mutation individuals show a single band consisting of the 60 and 64 bp fragments and heterozygous individuals show both the 124 bp undigested band and the band consisting of the 60 bp and 64 bp fragments (Fig. 2A, Supplementary Fig. S1).

For the mutation I1043L, an allele specific PCR was designed to identify the mutated allele. Four primers are used in a single PCR reaction: two common, external primers flanking the site of the mutation asymmetrically, a reverse primer specific for the wild type (susceptible) allele (having at its 3′OH a T) and a forward primer specific for the mutated allele (having at its 3′OH a C). In all reactions a common, control band of 352 bp is produced from the External_F and External_R primers. The ATC_R primer binds specifically to the wild type allele and amplifies with the External_F primer a fragment of 135 bp, while the CTC_F primer binds specifically to the mutated allele, pairs with the External_R primer and produces a 260 bp band. In heterozygotes both bands (135 and 260 bp) are observed (Fig. 2B, Supplementary Fig. S1).

Εach diagnostic is designed to detect the presence of one mutation, either the I1043M or the I1043L (Supplementary Fig. S2). Thus, both have to be applied to gain a clear picture about the presence of mutations associated with diflubenzuron resistance in field populations.

## Discussion

High levels of resistance against diflubenzuron, a very important mosquito larvicide for many places in the world (like Europe), was detected in *Cx*. *pipiens*, the major vector of WNV, in field populations from Ravenna, in Northern Italy. The presence and frequency of mutations at the aminoacid I1043 (I1043M and I1043L) in the *CpCHS* gene, an equivalent position to the BPU resistance mutation previously found in *P*. *xylostella*, was highly correlated with the resistance phenotype, as well as the ability of *Cx*. *pipiens* individuals to survive high dosages of diflubenzuron.

The introduction of I1056L in *D*. *melanogaster*, by the genome modification approach CRISPR/Cas9 coupled with homology-directed repair (HDR), showed a significant resistance phenotype (>20-fold) against diflubenzuron, albeit of lower intensity compared with the I1056M mutation, which conferred >15,000-fold diflubenzuron resistance^6^. *Cx*. *pipiens* individuals homozygous for either the mutations I1043M or I1043L survived at diflubenzuron concentrations exceeding the recommended WHO dose (i.e. 0.25 ppm of diflubenzuron, under optimum spraying conditions), which indicates that this resistance can dramatically affect diflubenzuron performance in the field. Indeed this has already been observed following quality control treatment, regularly conducted in the frame of the Emilia-Romagna mosquito control plan (data not published).

The identification of the resistant mutations with an apparent very strong effect on the phenotype, and the subsequent development of molecular diagnostics should substantially facilitate detection and monitoring of resistance in the field. This should delay the spread of the phenotype, particularly at this early stage, where the phenomenon seems very restricted in a small geographical area, based on available bioassay data around the globe, which have indicated a general susceptibility to diflubenzuron in both *Culex* and *Aedes* vectors^18,19^.

Should the molecular diagnostics developed in this study be successfully validated across geographical regions, they will be among the few available for monitoring insecticide resistance alleles, with direct links to operational significant levels of resistance in any mosquito species. Although a large number of molecular diagnostics are available to monitor field mosquito populations for the presence of insecticide resistance alleles (target site mutations or detoxification enzymes), the quantitative contribution of these molecular markers alone or in combination in resistance, and especially their operational significance, is often unclear^20^. Thus, many researchers would rather use bioassays (standard diagnostic protocols and/or intensity bioassays) to monitor the presence of resistant mosquitoes, their frequency in the population and the levels of resistance. However, bioassays have also practical limitations, such as the number of live mosquitoes required and integral variations. In this particular case, the application of the molecular diagnostics seems operationally more practical than bioassays. In addition the ability to detect heterozygotes, putatively susceptible and not detectable by bioassays based on the recessive nature of the resistant mutation in other species^6^, is very important for decision making, as it offers the opportunity to manage the phenotype at early stages.

The selection of resistance to diflubenzuron seems to be associated with the extensive use of this larvicide in the Emilia-Romagna region over the past ten years to control *Ae*. *albopictus* in road drains, a larval habitat exploited by *Cx*. *pipiens* as well. The potential for resistance selection could be high, since within only one year of systematic diflubenzuron applications, resistance increased from 32- to 128-fold in Ravenna.

The findings are of major concern for public health, as diflubenzuron is used in many places for the control of *Cx*.*pipiens* mosquitoes, transmitting West Nile and *Aedes* arbovirus vectors transmitting Dengue, Chikungunya and Zika. This is particularly the case in regions such as Europe, where neurotoxic insecticides have been banned from use in mosquito breeding sites. Screening of *Cx*.*pipiens* and *Aedes* mosquito populations from several geographical regions for possible resistant *CHS1* alleles must be conducted to guide appropriate resistance management strategies and ensure the sustainability of control interventions.

Due to the limited number of alternative mosquito larvicides available on the market, resistance is an important consideration for many countries, where diflubenzuron is in use, as the main mosquito larvicide, like Italy and several other European countries. Our finding raises further serious concerns regarding the reduction of reliable biocides for routine and emergency mosquito control, especially in cases of new invasive mosquito species. The development of additional mosquito larvicides and the re-consideration of old active ingredients, such as temephos might need to be strongly supported.

## Materials and Methods

### *Culex pipiens* populations

*Cx*. *pipiens* mosquitoes were collected during summer 2015 and 2016 as egg rafts in gravid traps in Ravenna, Italy, where mosquito control programs have been conducted with diflubenzuron for ten years. A *Cx*. *pipiens* laboratory strain (*Benaki*) that has not been exposed to insecticides for more than 20 years was included in the study as a reference susceptible strain^18^. Egg rafts were transferred to the laboratory and reared in climatic rooms at 28 ± 1 °C, 80% RH and 14:10 light:dark photoperiod.

### Bioassays with diflubenzuron

Standard WHO larval bioassays^21^ were performed using third-fourth instar larvae and diflubenzuron (DEVICE SC-15, diflubenzuron 13.9%, Arysta LifeScience, UK) doses ranging from 0.00015 to 0.468 ppm (dilutions made in water). For each concentration, four replicates of 25 larvae were used and emergence inhibition was measured daily until complete mortality or adult emergence was observed. Bioassays were conducted in climatic rooms as specified above. The data were subjected to probit regression analysis (Finney, 1971) using POLO-PC (LeOra Software POLO-PC, Berkeley, CA, USA).

### Genomic DNA extraction and analysis of chitin synthase sequences

Genomic DNA was extracted from individual *Cx*. *pipiens* adults using the Cethyl Trymethyl Ammonium Bromide (CTAB) method described in^22^. The DNA pellet was dissolved in 20 μl of sterile water.

A 825 bp fragment of the *Cx*.*pipiens* chitin synthase, spanning the 1043 position (numbering based on *Cx*.*pipiens* sequence), was amplified in a PCR reaction (25 µl final volume) using genomic DNA from individual mosquitoes, 0.4 μM primers (CHSseqF and CHSseqR) (Supplementary Table S1), 0.4 mM dNTPs, 2.5 µl of 10X buffer and 2 U of Kappa Taq DNA Polymerase (Kappa Biosystems, Inc., Wilmington, MA, U.S.A). The PCR conditions were 95 °C for 5 min followed by 30 cycles of 94 °C for 30 sec, 60 °C for 30 sec, 72 °C for 1 min and a final extension of 72 °C for 10 min. PCR products were purified using a PCR purification kit (Macherey Nagel, Dueren, Germany) and sent for sequencing (Macrogen Sequencing Facility, Amsterdam) using the primer 5′-ACGTTTGCGGGTGTGATGTC-3′.

### Functional characterization of I1056L using a genome-modified Drosophila line

For the generation of genome modified *Drosophila* flies, we used the strain y1 M{nos-Cas9.P}ZH-2A w*^23^, (nos.Cas9; stock no. 54591 at Bloomington Stock Center, Indiana, USA). For outcrossing and balancing we used strains yw and yw; TM3 *Sb e*/TM6B *Tb Hu e*, respectively. As positive controls in diflubenzuron toxicity bioassays, we used genome modified strain Px39^6^. All strains were cultured at 25 °C temperature, 60–70% humidity, and 12/12h photoperiod on standard fly diet.

The CRISPR/Cas9 system coupled with homology-directed repair (HDR) was used to introduce the mutation I1056L to the *krotzkopf verkehrt* (*kkv*) gene of *Drosophila melanogaster*, which is equivalent to the I1043L mutation detected on the chitin synthase 1 of *Cx*. *pipiens* (CPIJ016255, Vector base). The genomic engineering strategy followed was similar to the one described in Douris *et al*.^6^. Injections were performed with a plasmid mix containing 75 ng/μL of each of the RNA expressing plasmids gRNA444 and gRNA658^23^, to express gRNAs targeting the relevant genomic positions (Supplementary Fig. S3) and 100 ng/μL of a new specific donor plasmid (I1056L donor) for HDR. The donor plasmid was generated by site-directed mutagenesis using as template the donor plasmid used to generate the I1056M mutation^6^, in a site directed mutagenesis kit (Quick change II Site directed mutagenesis kit)(Agilent, California, USA), following manufacturer’s instructions, and primers MutI1056L F/R (Supplementary Table S1) to replace M1056 with L1056. The successful replacement was validated with sequencing. A schematic representation of the genome modification strategy is provided in Supplementary Fig. S3.

To screen for genome modified flies DNA was extracted from their tissues as in ref.^6^, using the DNAzol reagent (Thermo Fisher Scientific, Waltham, MA, USA) according to the manufacturer’s instructions followed by PCR amplification using the Kappa Taq DNA polymerase (Kappa Biosystems, Inc., Wilmington, MA, U.S.A) (1 unit), the ‘generic’ DroCHS forward primer (0.4μΜ) and the mutant allele specific DroCHS reverse primer (0.4 μΜ) (Supplementary Table S1). The PCR conditions were: initial denaturation at 95 °C for 5 min followed by 30 cycles of 30 s at 94 °C, 30 s at 60 °C, 40 s at 72 °C and a final extension step at 72 °C for 5 min. A 418 bp specific product is amplified only in the presence of mutated alleles. Screening was performed as in^6^; initially in pools of circa 30 G_1_ flies to identify lines where successful CRISPR/Cas9 plus HDR had taken place in G_0_ (injected) flies and later in individual G_1_ flies after outcrossing to yw. The progeny of positive G_1_ flies was crossed to flies carrying a balancer 3^rd^ chromosome (yw; TM3 *Sb e*/TM6B *Tb e*) and progeny flies carrying the I1043L mutation opposite to a balancer chromosome were intercrossed in order to generate homozygous lines bearing the genome modified allele. Allelic sequence was verified by sequencing the relevant PCR products (Macrogen Sequencing Facility, Amsterdam).

Second instar *D*. *melanogaster* larvae from control (nos.Cas9) and genome modified flies were transferred in batches of 15-20 in vials containing fly food supplemented with six different concentrations of diflubenzuron diluted in water (Dimillin [48% wt/vol diflubenzuron](Syngenta, Basel) causing mortality in the range of 0-100%. Each dose was performed in triplicates and control vials without insecticide were included. Dose-dependent molting and pupal eclosion were monitored for 10–12 days. LC_50_ values were calculated with Polo Plus (LeOra software).

### Molecular diagnostic assays

Following the identification of two mutations at the 1043 site (I1043M and I1043L) all sequences obtained from the screened individuals were aligned using the BioEdit software (Ibis Biosciences, Carlsbad, CA, USA) to identify the presence of single nucleotide polymorphisms spanning the 1043 site and design accurate molecular diagnostic assays.

#### Assay for the I1043M mutation

A PCR-RFLP diagnostic assay using the NlaIII (5′-CATG-3′) restriction endonuclease was developed to identify individuals carrying the I1043M mutation. A 124 bp PCR fragment flanking the I1043 site was amplified. The reaction was performed at a final volume of 25 ul, using 0.6 ul extracted gDNA, 2.5 μl of the 10X Kappa Taq Buffer A, 0.4 μM of each primer (Diagnostic I1043M_F and Diagnostic I1043M_R)(Supplementary Table S1), 0.2 mM dNTPs (Thermo Fisher Scientific, Waltham, MA, USA), 1.5U of Kappa Taq polymerase (Kappa Biosystems, Inc., Wilmington, MA, U.S.A) and water. The PCR reaction conditions were 5 min for 95 °C followed by 30 cycles of 30 sec at 95 °C, 30 sec at 60 °C, 30 sec at 72 °C and a final extension of 5 min at 72 °C. 5 μl of the PCR product were run on a 3% agarose gel to confirm successful amplification. The remaining 20 μl were digested with NlaIII (New England Biolabs, Ipswich, MA, USA) which selectively cleaves the resistant allele. The final volume of the reaction was 30 μl and included 5U of the restriction enzyme, 3 μl CutSmart Buffer (10X), 3 μl BSA (10 mg/ml) and water. The reaction was incubated for two hours at 37 °C followed by enzyme inactivation at 65 ^o^C for 20 min. The reaction products were run on a 3% agarose gel.

#### Assay for the I1043L mutation

An allele specific PCR was designed to identify individuals carrying the I1043L mutation, based on the method described in^24^. The PCR reaction of 25 μl final volume included 0.6 μl gDNA, 0.3 μM of the External_F, External_R and ATCspecific_R primers, 0.6 μM of the CTCspecific_F primer (Supplementary Table S1), 0.2 mM dNTPs (Thermo Fisher Scientific, Waltham, MA, USA), 2.5 μl Kappa Buffer A, 1U Kappa Taq polymerase (Kappa Biosystems, Inc., Wilmington, MA, USA) and water. The PCR reaction conditions were 5 min for 95 °C followed by 28 cycles of 30 sec at 95 °C, 30 sec at 68 °C, 1 min at 72 ^o^C and a final extension of 5 min at 72 °C. PCR products were run on a 2% agarose gel.

### Data availability

All data generated or analysed during this study are included in this published article (and its Supplementary Information files).

### Ethics statement

No animals were used to conduct this research. In general, all research activities respect fundamental ethics principles, including those reflected in the Charter of Fundamental Rights of the European Union (2000/C 364/01). The research is compatible with EU and international law, as a number of entomological monitoring activities is contacted worldwide and in Europe (European Mosquito Control Association, http://www.emca-online.eu). The molecular work was carried out in full conformity with Greek regulations consisting of the Presidential Decree (160/91) and law (2015/92) which implement the directive 86/609/EEC from the European Union and the European Convention. The experiments were carried out in certified facilities (license EL91-BIOexp-02) and the protocols have been approved by the Prefecture of Crete (license number # 27290, 15/12/2014).

## Electronic supplementary material

Supplementary Information
