# Author Correction: Striking diflubenzuron resistance in *Culex pipiens*, the prime vector of West Nile Virus

**DOI:** 10.1038/s41598-018-23059-1

**Published:** 2018-04-12

**Authors:** Linda Grigoraki, Arianna Puggioli, Konstantinos Mavridis, Vassilis Douris, Mario Montanari, Romeo Bellini, John Vontas

**Affiliations:** 1 Institute of Molecular Biology and Biotechnology, Foundation for Research and Technology-Hellas, 73100 Heraklion, Greece; 2 Department of Biology, University of Crete, Heraklion, 70013 Greece; 3 Medical and Veterinary Entomology, Centro Agricoltura Ambiente “G. Nicoli”, Bologna, Italy; 4 Azimut, Ravenna, Italy; 5 Department of Crop Science, Pesticide Science Lab, Agricultural University of Athens, 11855 Athens, Greece

Correction to: *Scientific Reports* 10.1038/s41598-017-12103-1, published online 15 September 2017

The Acknowledgements section in this Article is incomplete.

“We thank Yannis Livadaras (Foundation for Research and Technology Heraklion-Crete, Greece) and Dimitra Tsakireli (University of Crete, Greece) for their help with the functional assays.”

should read:

“We thank Yannis Livadaras (Foundation for Research and Technology Heraklion-Crete, Greece) and Dimitra Tsakireli (University of Crete, Greece) for their help with the functional assays.

This work was funded by the EC Horizon 2020 (European Union Framework Programme for Research and Innovation, INFRAVEC2), Grand Agreement 731060 and the LIFE CONOPS project “Development & demonstration of management plans against-the climate change enhanced - invasive mosquitoes in S. Europe” (LIFE12 ENV/GR/000466) co-funded by the EU Environmental Funding Programme LIFE+Environment Policy and Governance with the support of the Regional Health Authority of Emilia-Romagna.”

