# Supplementary material for: Striking diflubenzuron resistance in Culex pipiens, the prime vector of West Nile Virus
Source: Sci Rep. 2017 Sep 15;7:11699. doi: 10.1038/s41598-017-12103-1 (PMC5601912; doi:10.1038/s41598-017-12103-1)
Supplement: Supplementary file 1 — Supplementary Information [file 41598_2017_12103_MOESM1_ESM.pdf]

Striking diflubenzuron resistance in *Culex pipiens*, the prime vector of West Nile Virus

Linda Grigoraki[1,2], Arianna Puggioli[3], Konstantinos Mavridis[1], Vassilis Douris[1], Mario Montanari[4], Romeo Bellini[3], John Vontas J. [1,5]

[1] Institute of Molecular Biology and Biotechnology, Foundation for Research and Technology-Hellas, 73100 Heraklion, Greece

[2] Department of Biology, University of Crete, Heraklion, Greece, 70013

[3] Medical and Veterinary Entomology, Centro Agricoltura Ambiente ''G. Nicoli'', Bologna, Italy

[4]Azimut, Ravenna, Italy

[5]Department of Crop Science, Pesticide Science Lab, Agricultural University of Athens, 11855 Athens, Greece

*Corresponding author: J.Vontas, Fax and Phone number: (+30)2810394077; E-mail: vontas@imbb.forth.gr.

Supporting information

A)

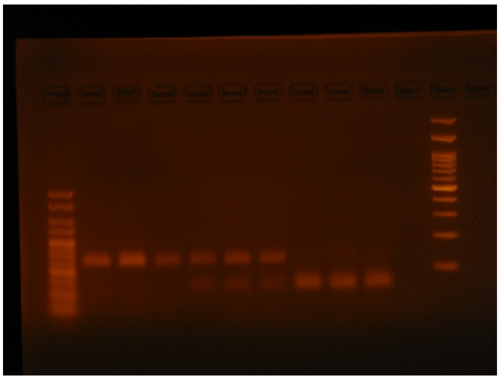

B)

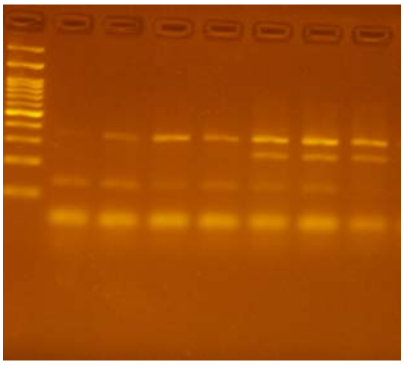

Supplementary Figure S1. Full length gels shown in Figure 2.

A) Full length gel shown in Figure 2A. B) Full length gel shown in Figure 2B. Note that the last 5 wells of this gel are depicted in figure 2B.

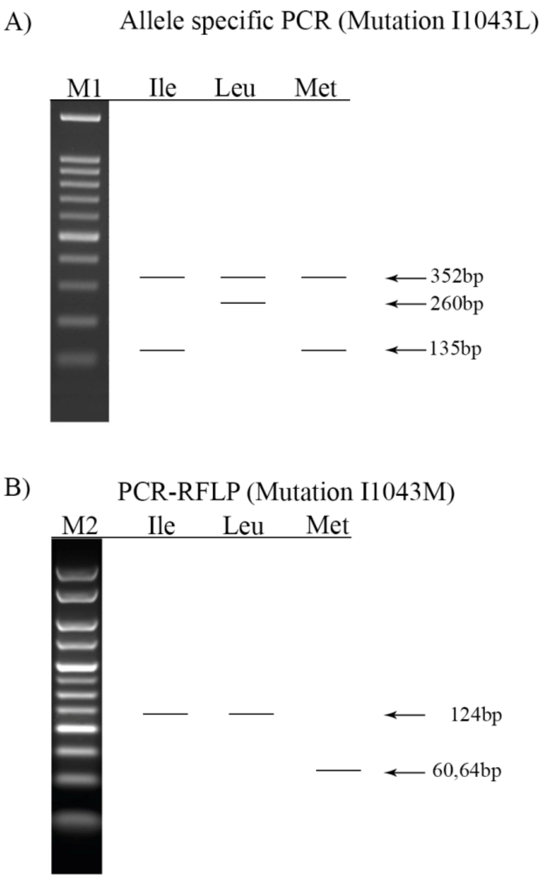

Supplementary Figure S2. **Schematic representation of the expected patterns for the two diagnostics**. (A: allele specific PCR for detection of the I1043L mutation and B:PCR-RFLP for detection of the I1043M mutation) in the presence of the wild type allele (I) and the two mutated alleles I1043M (M) and I1043L (L), which are associated with diflubenzuron resistance. M1: 100bp DNA ladder, M2: low molecular weight ladder (Hypperladder V, 25bp (Bioline,UK).

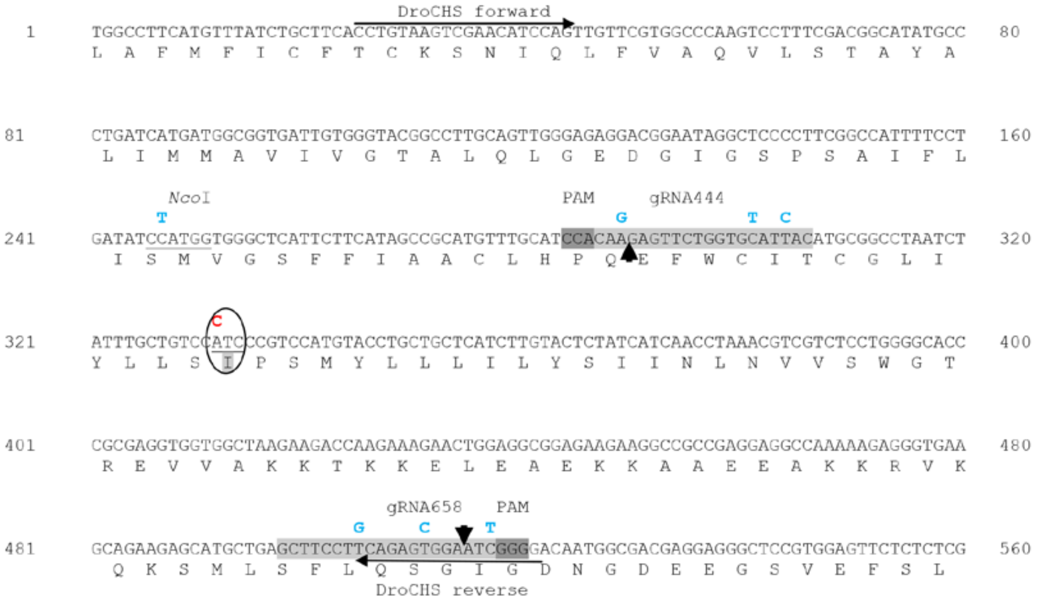

Supplementary Figure S3. **Genome modification strategy.** The nucleotide and amino-acid sequence of a 640bp fragment of gene *kkv*, flanking site I1056 of the *Drosophila melanogaster* amino-acid sequence are shown. Light gray areas correspond to the selected CRISPR/Cas9 targets (gRNA444, gRNA658) and letters in blue represent the synonymous substitutions introduced in the donor plasmid to avoid donor cleavage by Cas9 and abolish a *Nco*I site used for diagnostic purposes in [2]. Dark gray depicts the PAM (-NGG) triplets. Vertical arrows point to the CRISPR/Cas9 induced break points. The A→C transition at position 332 generating the (ATC→CTC) codon alteration that results in the I1056L mutation is marked with an oval shape. Horizontal arrows indicate the position of the DroCHS forward and reverse primers.

Primer	Sequence
Sequencing Chitin synthase	
CHSseqF	5’-CCGCGTTCAAGATTGACAACTGG-3’
CHSseqR	5’-TCCAGTAGGGGTTCGTCAGG-3’
Diagnostic PCR-RFLP I1043M	
Diagnostic I1043M_F	5’-GCCTGTCTCCATCCGCAAG-3’
Diagnostic I1043M_R	5’-CCCAGGAGACGACGTTCAG-3’
Allele specific PCR I1043L	
External_F	5’-GCAGTCCTTCGGCGATCTT-3’
External_R	5’-GAACAGTCCGGCGATGGATA-3’
ATC_ R	5’-AACAGCAAGTACATAGACGGGAT-3’
CTC_ F	5’-GGCTTGATCTACCTGCTGTCTC-3’
For screening genome modified Drosophila individuals	
DroCHS forward	5’-CCTGTAAGTCGAACATCCAG-3’
DroCHS reverse	5’-TCCCCAATTCCGCTCTGC-3’
For site directed mutagenesis of the CRISPR/Cas9 donor plasmid	
MutI1056L forward	5’-CCTAATCTATTTGCTGTCCCTCCCGTCCATGTACCTGC-3’
MutI1056L reverse	5’GCAGGTACATGGACGGGAGGGACAGCAAATAGATTAGG3’

Supplementary Table S1. Primers used in the study
